# Understanding DNA under oxidative stress and sensitization: the role of molecular modeling

**DOI:** 10.3389/fchem.2015.00043

**Published:** 2015-07-14

**Authors:** Elise Dumont, Antonio Monari

**Affiliations:** ^1^Laboratoire de Chimie, UMR 5182 Centre National de la Recherche Scientifique, École Normale Supérieure de LyonLyon, France; ^2^Université de Lorraine - Nancy, Theory-Modeling-Simulation, Structure et Réactivité des Systèmes Moléculaires Complexes (SRSMC)Vandoeuvre-les-Nancy, France; ^3^Centre National de la Recherche Scientifique, Theory-Modeling-Simulation, Structure et Réactivité des Systèmes Moléculaires Complexes (SRSMC)Vandoeuvre-les-Nancy, France

**Keywords:** DNA, photosensitization, photodynamic therapy, photochemistry, molecular modeling, energy/electron transfer

## Abstract

DNA is constantly exposed to damaging threats coming from oxidative stress, i.e., from the presence of free radicals and reactive oxygen species. Sensitization from exogenous and endogenous compounds that strongly enhance the frequency of light-induced lesions also plays an important role. The experimental determination of DNA lesions, though a difficult subject, is somehow well established and allows to elucidate even extremely rare DNA lesions. In parallel, molecular modeling has become fundamental to clearly understand the fine mechanisms related to DNA defects induction. Indeed, it offers an unprecedented possibility to get access to an atomistic or even electronic resolution. Ab initio molecular dynamics may also describe the time-evolution of the molecular system and its reactivity. Yet the modeling of DNA (photo-)reactions does necessitate elaborate multi-scale methodologies to tackle a damage induction reactivity that takes place in a complex environment. The double-stranded DNA environment is first characterized by a very high flexibility, but also a strongly inhomogeneous electrostatic embedding. Additionally, one aims at capturing more subtle effects, such as the sequence selectivity which is of critical important for DNA damage. The structure and dynamics of the DNA/sensitizers complexes, as well as the photo-induced electron- and energy-transfer phenomena taking place upon sensitization, should be carefully modeled. Finally the factors inducing different repair ratios for different lesions should also be rationalized. In this review we will critically analyze the different computational strategies used to model DNA lesions. A clear picture of the complex interplay between reactivity and structural factors will be sketched. The use of proper multi-scale modeling leads to the in-depth comprehension of DNA lesions mechanisms and also to the rational design of new chemo-therapeutic agents.

## 1. Introduction

DNA stability is essential to maintain cellular integrity of living organisms and avoid genetic mutations. Threats to DNA stability can be triggered by oxidative stress induced by the presence of metabolic reactive radical and oxygen species (ROS) or by photoreactivity. Oxidative and light-induced stress can have a very strong influence on the biological processes governed by DNA, as well as in the indirect activation or transduction of signal cascades, that may result in different malignant outcomes for the life of the involved cells (Salmon et al., 2004; Kujoth et al., 2005; Klaunig et al., 2010). Although, in the following we will focus more on OH^•^ induced pathways, it is worth mentioning that superoxide radical (0^2^_−_) is also connected to the induction of DNA lesions (Fridovich, 1995; Cadenas and Davies, 2000; Valko et al., 2007), as well as reactive nitrogen species (RNS) that are known for their potential carcinogenic activity (Pryor and Squadrito, 1995; Pacher et al., 2007; Sainz et al., 2012).

In contrast to photochemical degradation mechanisms, oxidative stress sources can also derive from endogenous processes and mechanism. Inflammatory conditions are a known source of ROS (Kamp et al., 2011), such as OH^•^, and remarkably ROS and free radicals are also exploited by the cells for signal transduction (Hamanaka and Chandel, 2010; Finkel, 2011), and hence are a key factor of cell metabolism. Moreover, ROS ultimately attacking DNA, may also derive as byproducts of the degradation of oxidized lipids or proteins, via the endocytosis process (Lim et al., 2004; Miyamoto and Di Mascio, 2014). In addition an other important source of endogenous ROS production, also related to neurodegenerative diseases, is due to the metabolism of dietary and biogenic amines (Chaiyen et al., 2012; Ramsay, 2012; Vianello et al., 2012; Repic et al., 2014). Indeed, this metabolic pathway performed by monoamide oxidase enzymes leads to the production of H_2_O_2_ and subsequently of OH^•^.

DNA is also constantly exposed to light, and hence efficient dissipative channels exist to hamper potentially dangerous photochemical pathways. Nevertheless, inherently stable DNA photoproducts have been reported and their photochemistry has been deeply analyzed (Sinha and Hader, 2002; Brash, 2015). Indeed, the correlation between DNA photo-lesions and the emergence of threatening mutations is now well accepted, and in particular the correlation between some types of skin cancers and uncontrolled sun exposure (Sage et al., 2005). Furthermore, the absorption range of native DNA can be significantly extended from the UVB region (where the constitutive nucleobases individually absorb) to the less energetic UVA because of the excitonic coupling within the macromolecular DNA structure, or thanks to the interaction with endogenous or exogenous chromophores, i.e., the photosensitization (Epe, 2012).

The cells response to the oxidative and photochemical stress normally results in an acceleration of protective mechanisms aimed to expel the stress enhancing factors. In the case of DNA lesions the activation of “repair” proteins, that may vary greatly in terms of specificity and efficiency, is invoked. The latter enzymes usually excise the damaged DNA area (Sancar and Sancar, 1988; Radzimanowski et al., 2013), therefore hampering any mismatch in the replication process. However some situations exist where the repair mechanisms fail to efficiently eradicate the lesions. In this case two scenarios are possible. On the one hand mismatches can occur during transcription, ultimately leading to mutations and eventually to carcinogenesis. On the other hand if the stress is too strong or the replication is impossible, for instance because it is blocked by interstrand cross-links, cells may induce their programmed death (apoptosis).

Apoptosis induction favored by the controlled production of DNA lesions has a very important therapeutic significance. Indeed, the formation of irreversible DNA lesions, and the interference with the replication process, has been and is currently exploited in the development of chemotherapeutic agents. For instance, we may cite the renown case of the cisplatin (Florea and Büsselberg, 2011), whose therapeutic effects are known since the 60's. More recently the cytotoxic action of other organometallic compounds, in particular Ruthenium complexes (Rademaker-Lakhai et al., 2004; Yan et al., 2005; Antonarakis and Emadi, 2010; Suss-Fink, 2010), has gained great attention in the development of novel chemotherapeutic drugs that are presently in clinical trial phase. Furthermore, since DNA lesions can be induced not only by ground state chemistry, but may also results from photochemical or photophysical activated energy- or electron-transfer processes the use of light-triggered therapeutic strategies and drugs appears promising in enhancing specificity of action and hence reduce unwanted side-effects. A particular attention should be paid to the light-induced activation of singlet oxygen that is promoted by opportune sensitizers such as porphyrins and lies at the heart of photodynamic therapy strategies (Dougherty et al., 1998; Pandey, 2000; Agostinis et al., 2011; Ethirajan et al., 2011).

From an experimental point of view, one relies on the use of techniques able to identify and possibly quantify the different lesions formed in DNA. Combination of mass-spectrometry with gas-phase chromatography is usually used to provide such informations both for isolated and cellular DNA (Frelon et al., 2000). Indeed, the difficulty in studying the reactions taking place in the complex macromolecular environment usually pushes toward the use of simple models, such as (artificial) mono- or dinucleotide. Even if such simplified techniques allow to infer and extrapolate valuable information on the nucleobase reactivity, still it is important to recall that the role of the DNA environment is almost totally neglected, while it may have huge chemical consequences. On the other hand NMR and X-ray spectrometry may provide extremely useful structures of lesioned DNA double-strand (Gold et al., 2014; Jain et al., 2014; Zalesak et al., 2014; Mutter et al., 2015). Even if in this case most often DNA inherent flexibility and complex dynamic are not properly taken into account, and hence the structural evolution and the role played by different lesions in driving such reorganization may be totally neglected. Obviously, to assess the response of DNA to UV/vis radiation the use of spectroscopic techniques is crucial. This can go from the standard and bench-scale use of optical techniques such as absorption, luminescence and electronic dichroism (Vorlickova and Palecek, 1974; Brabec et al., 1992; Ding et al., 2009) to more sophisticated time-resolved technique (Gustavsson et al., 2010, 2013; Vaya et al., 2012), allowing to follow the time-evolution of the different excited states. Furthermore, dichroism and spectroscopic titration are commonly used to infer the different and possibly competitive interaction modes between DNA and sensitizers (Carvlin et al., 1982; Wang et al., 2012; Lauria et al., 2014).

As this brief survey evidences, the induction of DNA lesions triggered by different sources of stress involves a very complex interplay between different phenomena taking place at molecular level, and having more general and systemic consequences. On the other hand, it is precisely that complexity that strongly suggests a multidisciplinary approach to tackle this non trivial problem, in particular combining adequate spectroscopic techniques with state-of-the-art molecular multiscale modeling. Molecular modeling is invaluable in providing atomistic or even electronic scale description of the involved phenomena. For instance, molecular dynamics techniques allow to assess for the structural deformation of lesioned DNA and its time evolution, as well as to infer the existence of different stable interaction modes with smaller sensitizers. The ground- and excited- state behavior and the reactivity, for instance in terms of free energy profiles along reaction coordinates, are described by using hybrid quantum mechanics/ molecular mechanics (QM/MM) methods allowing to take into account the role of the environment (Senn and Thiel, 2007, 2009; Meier et al., 2013; Monari et al., 2013). QM/MM also allows to validate the experimental use of simpler and more homogeneous model systems (Ding et al., 2009). The growing computational power experienced in the latter years, as well as the presence of more and more efficient algorithms and codes allows to tackle more and more complex problems and environments, providing an unprecedented comprehension of biologically relevant mechanisms.

In the present review we will present some examples of the molecular modeling of DNA under external stress, both concerning ground-state and photochemical pathways, underlying the fundamental questions that may be answered by a proper simulation and modeling.

## 2. Modeling of DNA oxidative stress

DNA oxidative stress is dominated by a combinatorial radical chemistry, in biological media hydroxyl (HO^•^) and peroxyl (HOO^•^) radical being by far the principal players. However, other non-radical reactive chemical moieties can play a role and have a strong biological influence: ^1^O_2_, H_2_O_2_, alkylating agents, low-energy electrons, and each of them has been tackled by simulation means.

### 2.1. An ubiquitous first step: Hydrogen abstraction

**Figure 1:**
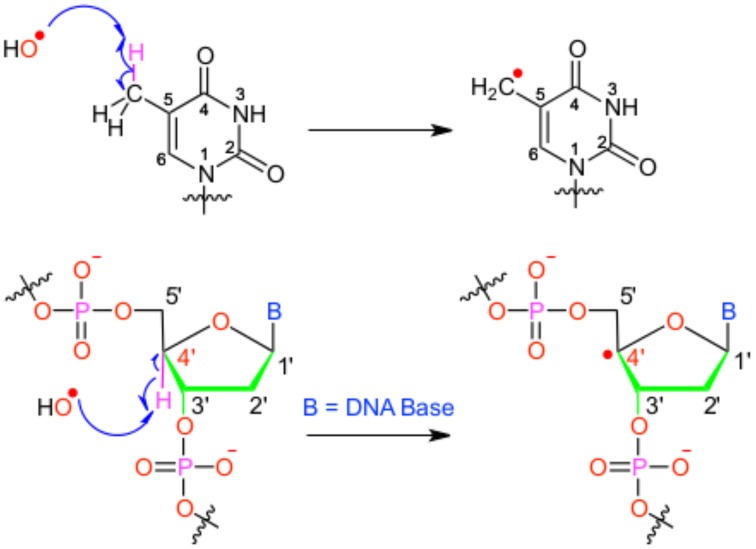
**Scheme of the different possible oxidative attacks by OH∙ on DNA nucleobase or backbone sugar moiety**.

We will hereby focus on radical induced ground-state reactivity, and mostly on the hydroxyl radical HO^•^, as its study exemplifies the methods employed in DNA damage molecular modeling. The hydroxyl radical is prone to abstract an hydrogen, with subsequent production of one water molecule and an highly reactive radical specie embedded in the biological macromolecule (see Figure 1); hence, this first step may also constitute the initiation step in radical chain reactions.

**Figure 2:**
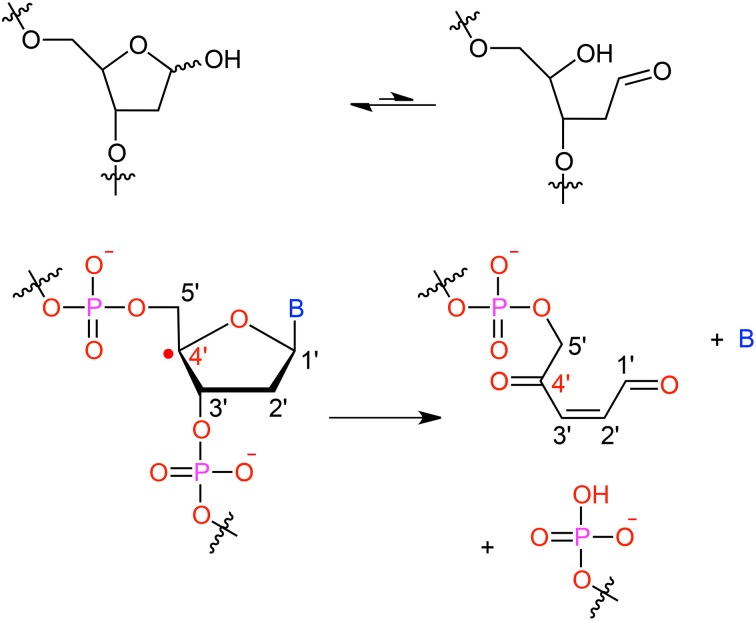
**Evolution to abasic site and DNA strand scission following oxidative attack on the DNA sugar moiety**.

It has been shown that for a given double-strand DNA fragment (ds-DNA), an hydrogen uptake can occur at many positions, on the nucleobase (Cadet et al., 1999) but also on the sugar moiety (Pogozelski and Tullius, 1998), with a ratio of 9:1. Another reactive pathway activated by the hydroxyl radical is the direct addition of HO^•^ onto the an ethylenic position of a given nucleobase B, thus forming *in situ* a radical entity [B–OH]^•^. Once again [B–OH]^•^ may undergo further fragmentation or evolve toward the formation of a peroxyl nucleobase. Thus HO^•^ has a deleterious and versatile, chemical outcome with many possible subsequent fragmentation patterns. For instance, as schematized in Figure 2, a possible evolution may lead to the generation of abasic sites, or to the oxidative strand scission of nucleic acids (Cooke et al., 2003). Abasic lesions are characterized by oxidative pathways that lead to the disruption of the base pyrimidine or purine kernel, leaving only the sugar as part of the strand. On the other hand strand scission, mostly affect the sugar and the backbone (Figure 2) and ultimately results in a breaking of the strand continuity. If the previous lesions may easily be recognized and repaired by specific enzymes, also more dangerous lesions, particularly refractory to the repair may be produced (Bergeron et al., 2010). The former are produced by the attack of an oxidized nucleobase onto a vicinal one (either situated on the same strand or on the opposite strand), this gives rise to intra- or interstrand cross-links respectively. This particularly complex and significant case will be dealt to in Section 2.3.

Modeling has been early invoked to provide a rationale, and ultimately predict, the preferential hydrogen abstraction sites: indeed, because of its general high reactivity, the hydroxyl radical selectivity is governed by the accessibility to the solvent. The latter is an information reliably estimated by bioinformatics approaches such as the computation of Solvent Accessible Surface Area (SASA). Tullius et al. have exploited this approach to rationalize the HO^•^-induced DNA strand breaking (Balasubramanian et al., 1998), hence providing a clear-cut structural basis to rationalize the abstraction site preference. Indeed the authors have clearly shown the existence of a very good correlation between the reactivity of the different sites and their solvent. The C5 carbon atom was indeed found to contribute to the total cleavage by 57%, the C4 position accounting for the 22% and the C3 for the 17%. The most reactive C5 position on the other hand shows a solvent accessible area of 46%, while the C4 and C3 are characterized by 28 and 14% accesible surface, respectively. Furthermore, this rather simple approach may easily be generalized to preview the reactivity in presence of diffusion controlled reactants and may be extended to the investigations of RNA reactivity and repair processes. Monte-Carlo simulations of site-specific radical attack to DNA bases have also proved their usefulness (Nijkoo et al., 1999; Bulent et al., 2008), with the advantage to go beyond the single-structure and static description. On the other hand, the almost immediate estimate of SASA paves the way toward massive parallel-sequencing-based hydroxyl radical probing of RNA accessibility (Kielpinski and Vinther, 2014).

The hydrogen abstraction is the key limiting step of the whole damage process (Regulus et al., 2007; Nikitaki et al., 2015), and its in-depth understanding can take full profit from quantum mechanics calculations: the latter are most often rooted in density functional theory (DFT), which allows to discriminate the most stable radical centers. Most particularly, DFT can easily be used to calculate the carbon–hydrogen bond dissociation energy (BDE) on different nucleobase and sugar potential reactive sites. This description allows to go beyond the simple solvent accessibility, indeed a lower BDE will be recognized as a crucial factor favoring the reactivity of the specific site.

For instance, DFT confirms that hydrogen abstraction for pyrimidine nucleobases, i.e., cytosine and thymine, occurs preferentially on C5 positions and on the corresponding methyl group (C5 m), not only because of solvent accessibility but also due to electronic favors (Ji et al., 2005; Frances-Monneris et al., 2013; Yadav and Mishra, 2013). Interestingly enough, while most natural occuring nucleobases lead to the formation of π-radicals upon H abstraction, the substitution of the carbonyl oxygen with sulfur induces the formation of the quite rare σ-radicals (Besic et al., 2001; Vianello and Maksic, 2003; Gomzi, 2011).

It is remarkable that a sound agreement can be achieved between experiment and theory even when calculations on model, isolated moieties are performed (Bera and Schaefer, 2005). More recently, Papiez et al. have employed Car-Parrinello molecular dynamics (CPMD) to describe the hydrogen abstraction on a solvated guanine (Wu et al., 2004; Abolfath et al., 2012). This work nicely underlines the contrast between the static approaches where temperature effects and hence thermal fluctuations are neglected and the dynamic picture. Indeed while in vacuum the hydrogen abstraction occurs at around *t* = 0.07 *ps*, water solution slows down the reaction rate roughly by a factor of two (*t* = 0.12 *ps*). The hampering effect of the water environment is due to thermal fluctuation of the solvent molecules, that are also highly organized because of hydrogen-bond networks, ultimately resulting in a strong disturbance of the OH· motion. The extension of this protocol to a ds-DNA model would constitute an interesting perspective. However reaching such a goal will require describing most of the system at the molecular mechanics (MM) level, while the reactive center (i.e., the nucleobase) should be described quantum mechanically (QM). Furthermore, water molecules close to the nucleobase should be included in the QM partition, but due to water high mobility one has to take into account the fact that some solvent molecules may move out from the significant QM partition while others may enter. Such schemes, known as adaptative QM/MM, have been pushed forward over the last years (Nielsen et al., 2010) but are for now applied only to model reactions. An interesting alternative for large scale molecular modeling could rely on the use of *reactive* MD, which has been realized relying on the ReaxFF force field (Abolfath et al., 2011).

However, QM/MM-MD simulatons, e.g., in a Car-Parrinello framework, have nowadays been successfully performed both for reactivity in DNA double strands and in solvated nucleoside (Abolfath et al., 2012; Garrec et al., 2012). Compared with reactive MD force-fields and previous calculations, these studies have the advantage to exploit a less-parameterized density functional. Furthermore, they also provide a more direct comparison of free-energy profiles between an isolated system and a DNA fragment. Indeed, it is worth emphasizing that DFT has been employed in a near-exclusive way not only for ground-state reactivity, but also for calculating eletronical vertical excitation (Durbeej and Eriksson, 2003). More recently, several works have also described *photo-induced* hydrogen abstraction (Szabla et al., 2015), resorting to a multi-reference description of the electronic density through complete active space self-consistent (CASSCF) or second order perturbation theory (CASPT2) approaches. Remarkably, in the previous contribution the authors identified a reactive conical intersection joining the S_0_/S_1_ of β-2′-deoxycytidine. Although, from the S_1_ minimum the system should overcome a rather large barrier of 0.59 eV to reach the conical intersection, the excess vibrational energy at Franck-Condon geometry safely allows for its crossing and hence justify the active photochemistry. Even if multi-configurational methods can in principle be used within a QM/MM-MD framework, they are mostly restricted to the treatment of excited states due to their efficiency in the description of non-adiabatic potential energy points (conical intersection) and to the computational bottleneck that limits the time-scale of the MD trajectory to hundreds of fs (Szabla et al., 2015). However, unlike for other systems or reactions, the exchange-correlation density functional dependence is less prononouced for the study of DNA and nucleobase hydrogen abstraction reactivity, and different functional classes usually provide the same insights. In contrast, the inclusion of a dispersion is particularly important as it was evidenced for instance by the Bickelhaupt's group (Poater et al., 2011).

### 2.2. Probing the role of the environment for single-nucleobase reactions

When coming to DNA reactivity and damage generations, experiments are performed on isolated nucleobases, small DNA fragments (trinucleotides), longer oligonucleotides or even larger ds-DNA structures. It is now well recognized that reactivity in model systems can largely differ due to the geometrical constraint imposed within a ds-DNA and the strongly heterogenous environment. Providing a most direct comparison between reaction profiles for isolated moieties vs. nucleobases embedded in a complex macromolecular environment allows to pinpoint the structural and electrostatic factors that can dramatically tune or reserve the intrinsic reactivity of nucleobases. Many elementary lesions in DNA subunits have been first targeted based on QM studies on isolated fragments (Duncan Lyngdoh and Schaefer, 2009).

Predictions of the reactivity order between different nucleobases can indeed be assessed on model π-stacked systems (thymine propensity to form lower triplet states or dimerize (Durbeej and Eriksson, 2003), lower ionization potential of guanine, …). Both DFT and (multireference) post Hartree-Fock approaches can be employed, (e.g., on the guanine radical cation Hutter and Clark, 1996; Sponer et al., 2001; Jurecka et al., 2006). DNA reactivity has been a field of investigation for conceptual DFT, with derivation of DFT-based descriptors to rationalize intrinsic trends in DNA reactivity (Labet et al., 2014). Yet, this supposes that reactivity is governed only by electronic factors, neglecting the influence of the strongly heterogeneous environment, and the fact that the flexibility of short oligonucleotides can induce noticeable deviations from the canonical B-DNA parameters.

Cluster models where a reaction profile is obtained employing hybrid QM/MM (Barnett et al., 2006) or QM/QM′ approaches (Cauët et al., 2013; Ceron-Carrasco and Jacquemin, 2013; Jacquemin et al., 2014) provide a first step toward more realistic systems. However in these models dynamics is neglected and implicitly such schemes assume that reactivity occurs with no distortion of the B-helix (extremal nucleobases are kept frozen). Furthermore, even larger deviation of the ideal B-DNA structure can be induced by the lesions themselves, with consequences that can be crucial for further reactivity or for the repair ratio.

Structural and dynamic changes of an oxidized abasic site in ds-DNA have been studied by molecular dynamics to unravel representative conformation(s) of the double-helix and infer the role played by structural modifications on the DNA chemical reactivity (Chen et al., 2008; Patel et al., 2013). Indeed it has been shown that abasic sites shows a great conformational flexibility (Patel et al., 2013), and most notably MD has unambiguously shown that abasic site is much better stabilized in the B-DNA double strand when coupled with a cytosine. Indeed in this case the lesion is accomodated in the duplex and stabilized by a pair of hydrogen bonds, in contrast purines nucleobases may at most form one hydrogen bonds and as a consequences the lesions constantly flip in and out of the duplex. The blocking of the abasic site inside the B-DNA by cytosine has a strong consequence on reactivity since it can favor reactive conformations leading to interstrand cross-links.

Indeed, MD simulations provide a starting point for tackling the reactivity, within the framework of hybrid QM/MM-MD simulations. A decade ago Parrinello and coworkers rationalized the role of the B-DNA (Gervasio et al., 2004) on the reactivity of the guanine radical cation. This pioneer work constitutes a first probe of a reversal for the stability of the GC^+^:C vs. G(−H)C :C(H)+ couple in B-DNA, which is due to the electrostatic interaction with the negatively charged backbone as well as to the different geometrical structures adopted by the pair.

QM/MM-MD simulations of DNA reactivity are becoming more and more popular, with several choices concerning the biais techniques (metadynamics Gervasio et al., 2004, umbrella sampling, thermodynamic integration Garrec et al., 2012) to sample the free energy reaction profile along one or several reaction coordinate(s).

The next subsection is devoted to theoretical studies focusing on reactions implying two nucleobases, where the distortion of the DNA structure is even larger and hence a proper dynamic description of the reaction becomes crucial.

### 2.3. Bridging two nucleobases: Structure and reactivity

After the initial radical hit, modified nucleobases (X), such as oxidized AP, see Figure 2, can be formed which are prone to form covalent intra- or interstrand cross-links (ICL) with vicinal nucleobases. One often lacks the knowledge of a proper experimental structure of the damage to ascertain the conformation of the reactant, or to probe the final structure of the product (Patel et al., 2013).

Formation of ICLs obeys a multistep pathway that can be studied based on model systems (Labet et al., 2008; Sviatenko et al., 2012). But the study of ICLs also differs from single-nucleobase lesions since the B-DNA structure is much strongly impacted by the formation of this kind of defect, which could be in line with the fact they are refractory to repair.

The formation of ICLs can be formally written as X → B, where X denotes the modified (oxidized) nucleobase and B one of the four canonical nucleobases (Figure 2). Their modeling covers two central aspects:

1. The reactivity to situate the barrier to covalently bind two nucleobases together. This can rely on DFT calculations performed on dinucleoside monophosphate, in gas-phase or with implicit solvation. Alternatively the reactivity can be treated within a B-DNA fragment, to account notably for the helical embedding. The barrier to overcome to bridge up two nucleobases together is strongly dependent on DNA conformation and on the nucleobases themselves. However this dependence is non trivial, and attacks corresponding to strand offset have also been reported.
2. The structural consequences of the formation of such bonds, and in particular the free-energy necessary to induce the helical deformation. In this case molecular modeling can palliate the lack of experimental structures. In particular modeling provides starting conformations of the reactant, but perhaps more importantly, provides a computationally efficient way to build up in silico the structures of a given modified oligonucleotide.

The latter point is fundamental in the absence of NMR or X-ray structure even for the most commonly formed intra- and inter-strand oxidatively-generated lesions. Also, from a modeling perspective, the typical timescale for the reorganization of a 12 base-pair oligonucleotide bearing a central defect, poses a central challenge since it spans tens of nanoseconds, to be compared to the current limitations of 10–100 ps for QM/MM-MD schemes. The identification of suitable reaction coordinates and collective variables describing the process will allow the use of rare-event accelerating methods such as meta-dynamics, however the problem is still far from being trivial. Their inherent complexity also explains why the modeling of this class of lesions is much less developed, despite their strong biological significance.

One important breakthrough in the study of intrastrand-cross links has been recently performed using Car-Parrinello QM/MM molecular dynamics (Garrec et al., 2012). In this paper the authors have performed thermodynamic integration to unravel the free-energy profile of the reaction between cytosine and a nearby C5m-thymine radical leading to the so called G[8,5-Me]T lesion. The geometrical constrains imposed by the B-DNA environment helps in keeping the reactant in an optimal π-stacked conformations, and as a results the process is characterized by a relatively low activation energy (about 10.0 Kcal/mol) on the other hand isolated nucleotides shows a rather larger deviation from the ideal conformations and as a result the activation free energy goes up to about 70.0 Kcal/mol. Furthermore, the relatively small disruption of the ideal B-helix structure induced by G[8,5-Me]T could also explain the observed low reparation rate.

All in all it clearly appears that the modeling of ground-state DNA reactivity is an extremely complicated problem requiring the correct equilibrium between a proper description of the electronic factors and a suitable sampling of the conformational space of a very flexible macromolecule. To tackle the latter problems some techniques developed in particular in the field of the enzymatic catalysis (Garcia-Mesenguer et al., 2013; Zinovjev et al., 2013; Zinovjev and Tunon, 2014) should be considered both to define proper reaction pathways and to perform a good sampling compatible with the reaction time-scale. On the other hand the problem of an accurate sampling of the conformational space or of the definition of suitable collective variables is less dramatic when dealing with excited-state phenomena, i.e., photochemistry and photophysics, even if still the coupling of electronic factors with vibrational and dynamical ones cannot be underestimated. These aspects will be the focus of the next section.

## 3. Interaction between DNA and UV radiation

DNA is constituted by π-stacked nucleobases that can be regarded as chromophores and whose absorbance is mostly confined in the UVB regions of the spectrum. The absorption region capable of producing DNA lesions can be enlarged for instance by photosensitization, i.e., by the interaction with endogenous or exogenous compounds (Epe, 2012; Cadet and Wagner, 2013).

### 3.1. Direct UV absorption and decay

Even if UVB wavelengths are normally filtered by the ozone layer, and efficient deactivation pathways of the nucleobases excited states exist, DNA lesions resulting from direct photoactivation are usually reported and are considered as extremely dangerous for their potential carcinogenicity.

The photochemical pathways leading to the formation of the cyclobutane thymine dimer (*T* <> *T*) and of the 6-4 photoadducts [(64)-PP] in the case of stacked nucleobases has been the subject of interesting combined theoretical and spectroscopic study (Banyasz et al., 2012). After a thoughtful validation of TDDFT techniques by comparison with high-level CASPT2 techniques the authors efficiently analyze the differences between the paths leading to the two main photoproducts [*T* <> *T* or (64)-PP]. In particular in the case of *T* <> *T* dimerization, after absorption at 5.36 eV, an efficient and barrierless path in the first ππ^*^ excited state leads to a short lived minimum at 4.45 eV connected to the conical intersection (CI) region. Furthermore, the CI is characterized by a very short inter-monomer distance, most notably the C5-C5′ and the C6-C6′ distances have been found to be of 2.5 and 2.06 Å, respectively, hence the topology of the CI clearly favors dimerization. Notably, no sterical hindrance or electrostatic effect due to the backbone are observed. On the contrary the formation of the (64)-PP adducts, and in particular of the key oxetane intermediate, involves the population of a charge transfer (CT) state. The CT state involves both thymines and is found at 5.50 eV. From Franck-Condon region the excited states may relax toward a very short living minimum that efficiently evolves toward the oxetane conical intersection region. Once again, the geometrical features of this conical intersection favor dimerization, in particular very short intermonomer distances are found (C6-C4′ 2.46 Åand C5-O8′ 1.65 Å). Note that environment effects are crucial since the backbone charges efficiently stabilize the charge-separated state compared to the gas-phase situation, and hence strongly favor its population. Furthermore, a barrier in the CT state potential energy surface, and probably due to dynamical solvent effects, needs to be overcome. This fact supports the evidence that although the CT state is reachable upon UVA irradiation, shorter wavelengths excitation are needed to allow the system the excess kinetic energy.

Recently, more complex photochemical mechanisms have been taken into account (Esposito et al., 2014). Once again combining spectroscopy and modeling, the study of a trinucleotide of sequence TCG allowed to gain insight into the competition between the formation of cyclobutane pyrimidine dimer (CPD) and (64)-PP adducts. Interestingly the role of the methylation of the 5′ position of guanine has been shown to be far from innocent in dictating the relative ratio between the CPD and (64)-PP. Indeed, the authors found that upon methylation the ratio between the quantum efficiency for the CPD production versus the (6-4)PP one (φ_*CPD*_∕φ_(6−4)*PP*_) goes from 1.52 to 3.68. The important increase of the selectivity toward CPD has been rationalized by combining QM and MD calculations and ascribed to the sugar puckering which modulates the stacking arrangement of the reactants. Indeed, methylation strengthens interaction with flanking T and globally stabilizes conformers that are more reactive toward CPD formation. Since C5 position metyhylation of guanine is known to play important role in epigenetic gene regulations and is found as hot-spot in skin cancer the biological relevance of these findings are evident. The influence of the double helical pairing has also been taken into account, globally confirming the previous results.

The same group (Vayá et al., 2012) has also revealed the role of the DNA structural organization in dictating its spectroscopic and photochemical properties. Indeed the exciton coupling of the nucleobases is reflected in an energy- and charge-transfer along the double helix that is reflected in the fact that DNA lesions hot-spot depend on the nearby base sequence and are not randomly distributed. From a photophysical point of view an interplay between charge and energy transfer is observed. Still it will be important to determine the sequence effects favoring charge-separation and charge-recombination.

Barbatti and Lischka have reported a systematic study of the deactivation pathways for the four DNA nucleobases (adenine, thymine, guanine and cytosine) as well as for uracil (Barbatti et al., 2010). The use of non-adiabatic state-hopping molecular dynamics (Barbatti, 2011) has allowed the authors to give clear insights on the deactivation pathways and to reveal two distinct behaviors between purines and pyrimidines. Indeed, adenine and guanine have a relatively simple photodynamic, characterized by a rapid evolution from the ππ^*^ up to the conical intersection, where they are funneled back to the *S*_0_ ground state. This simple mechanism is also reflected in the extremely fast deactivation with an estimated excited state life time of 0.28 and 0.77 ps for guanine and adenine, respectively. In contrast, for pyrimidines a much more complicated situation is found with the competition of two channels: one evolving to the ππ^*^ conical intersection and the other one toward the nπ^*^ crossing seam. The ratio between the two channels depending on the bases, for instance in cytosine the direct ππ^*^ path is not activated at all. The more complex photochemistry of pyrimidine bases, also due to the presence of low lying ππ^*^ states with flatter potential energy surfaces, is reflected in the longer life-time of excited states spanning values from 0.53 to several ps.

Robb group (Groenhof et al., 2007) also reported a computational study of the deactivation pathways of a cytosine-guanine base-pair both in gas phase and embedded in a DNA environment. Their QM and QM/MM multiconfigurational calculations revealed that the deactivation pathway starts with a proton transfer, that is followed by a radiationless decay leading to an extended conical intersection seams. Indeed, the particular topology of the conical intersection systems may lead to a number of crossings between the first excited and the ground state, that may ultimately lead to a diabatic blockage. After the crossing to the ground state the proton is transferred back and in a majority of situation the initial conformation is restored. Interestingly, comparing QM and QM/MM calculations, the authors conclude that the Watson-Crick pairing keeping the base pair closer together enhanced photostability minimizing the fraction of trajectory ending up in a different tautomeric state compared to the initial configuration.

Novel strategies to prove the photostability of DNA nucleobases start emerging, notably very recently Garavelli's group reported the modeling of near UV 2D-electronic spectroscopy as a tool to characterize the double excited, multi excimer and charge-transfer states that may be responsible of the ultrafast deactivation dynamics. These advancements could pave the way toward a better interplay between theory and modeling to give an atomistic level description of the different dynamic phenomena (Nenov et al., 2015).

Although, extremely efficient DNA relaxation pathways may lead to a drawback evidenced by the works of Gonzalez's group that have underlined a femtosecond intersystem crossing taking place in the case of cytosine (Richter et al., 2012). The explicit inclusion of spin-orbit coupling in the calculation of the hopping probability has evidenced a non-negligible population of the triplet state manifold, indeed after 140 fs the *T*1 state has an important population of 10%. This striking result, even if it neglects the environment, is extremely important from a photophysical point of view, since it illustrates a situation in which the quasi-degeneracy between triplets and singlets compensates the moderate, but non-negligible, value for the spin-orbit coupling. Moreover, from a more biological point of view, if confirmed in the DNA embedding it may open the way to the population of triplet states, that could be responsible of indirect production of DNA lesions.

### 3.2. DNA photosensitization

In DNA photosensitization the absorption of light and the subsequent photochemical pathways are mediated by an interacting chromophore. By definition in the case of sensitization the chromophore needs to be recovered inaltered at the end of the process, acting in an almost catalytical way. This excludes covalent interactions with DNA that characterize the action of important antitumoral drugs such as the cisplatin.

**Figure 3:**
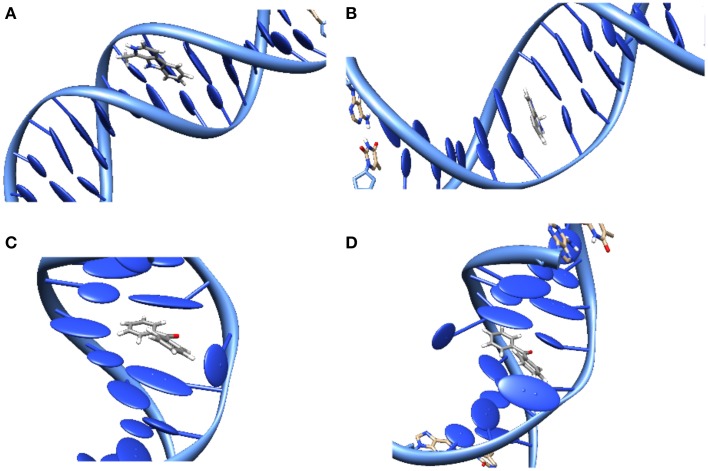
**Representation of the main interaction modes between DNA and sensitizers**. **(A)** Groove-Binding; **(B)** Intercalation; **(C)** Insertion; **(D)** Double Insertion.

The study of DNA photosensitization is complicated by the fact that one should be able to achieve a good knoweldge of different phenomena. First of all, a clear picture of the structure of the DNA/sensitizers aggregate is compulsory. Usually three main modes of interaction have been characterized for canonical B-DNA (Zeglis et al., 2007), see Figure 3:

- **Groove Binding**. In which the sensitizer lies close to the DNA grooves, and stabilization is mostly driven by electrostatic interactions with the negatively charged backbone. Note that usually a rather important selectivity is evidenced between the minor or major groove, and that the double-helical structure is only scarcely distorted.
- **Intercalation**. In which the sensitizer slips between two base pairs. The stabilizing interactions are mostly due to dispersion and π-stacking with the DNA nucleobases, and hence this mode is predominant for rather large, planar, conjugated structures. The DNA helix is slightly more perturbed since an intercalation pocket needs to be created.
- **Insertion**. In which the sensitizer ejects one of the bases from its Watson-Crick pairing, taking its place in the helix. Conjugated systems with non-collinear fused rings, i.e., mimicking the pyrimidine geometries, are the most likely to prefer insertion over intercalation.

Moreover the three interaction modes can most often coexist, with a population ratio strongly dependent on the environmental factors such as the DNA sequence or the salt concentration. As a consequence, X-Ray resolved structures are quite rare, and structural determination becomes crucial, requiring a proper modeling that may confirm more indirect experimental observation such as the ones based on circular dichroism.

On that context we should cite the extensive molecular simulations performed by the Lavery group using molecular dynamics and different sampling techniques such as metadynamics and umbrella sampling (Wilhelm et al., 2012). The authors were able to unambigously determine two stable states for the binding of Daunomycin drug to DNA: minor groove binding and intercalation. Furthermore, they were able to recover the free energy profile along the pathway connecting the two modes. A thermally accessible low barrier of about 6.5 kcal/mol was indeed found to separate the two stable modes, with intercalation being about 2.0 kcal/mol more stable. Interestingly, also an intermediate state was found with the drug only partially intercalated while DNA is strongly bended away from the drug. This intermediate state, sometimes called semi-intercalation, is also found in a number of other drugs or even in the case of minor-groove binding protein. In the case of daunomycin, however, the particularity lies in the fact that it is only connected to a very shallow minimum in the free energy profile, and hence does not constitute a kinetic blockage for the full intercalation.

Also the interaction between simple organic molecules and DNA should be described most carefully, which necessitates the resort to molecular dynamic simulations to sample the relevant possible conformations. A particularly striking case is the one of benzophenone, a paradigmatic sensitizers (Cuquerella et al., 2012), for which no experimental structure has ever been reported for the interaction with DNA. Indeed, by analyzing the molecular dynamics trajectory only 2 stable interaction modes were evidenced (Dumont and Monari, 2013), one of them was the usual minor-groove binding, but the second one was a novel mode called “double insertion” (Figure 3). In this mode the sensitizer, benzophenone, ejects both the bases constituting the Watson-Crick pair and take the place of both in the stacked structure. The use of the so-called non covalent interactions (NCI) technique to visualize non-covalent interaction (Johnson et al., 2010) allowed to pinpoint the emergence of a very strong hydrogen-bond between the ejected base and the phosphate of the backbone as one of the factors explaining the stabilization of the double-insertion mode, even in presence of a strong helical deformation. Furthermore, the calculation of induced circular dichroism, using QM/MM methods coupled to the MD simulation, evidenced a clear spectroscopic signature for the double-insertion in the UVA region that could allow for an easy experimental confirmation (Dumont and Monari, 2013). Indeed, while the minor groove-bound benzophenone does not show any induced circular dichroism spectra, the double-inserted mode presents a well resolved negative band at 350 nm, corresponding to the nπ^*^ excitation. Since this band belongs to the UVA and is far from the native DNA absorbing region it is supposed that it could allow for a quite straightforward discrimination of the interaction modes. Recently and independently the double-inserted mode has been also evidenced in the case of copper complexes interacting with DNA (Galindo-Murillo et al., 2015) using very long scale molecular dynamics up to the μ*s* scale.

Photosensitization involving the excitation of the chromophore, and the subsequent excited-state interactions with the nearby nucleobases, the influence of the environment on excited state properties is crucial and should be thoughtfully taken into account. Indeed the optical properties of many chromophores can be modulated by the environment, one of the most well known cases being the light-switch effects (Friedman et al., 1990), i.e., the activation of luminescence induced by DNA for the Ruthenium complex [Ru(dppz)(bipy)_2_]^2+^ (bpy = 2,2′-bipyridine; dppz = dipyridophenazine). Indeed the latter complex happens to be dark in water, weakly luminescent in non protic solvent like acetonitrile and strongly luminescent in water. One of the first rationalization of this phenomenon was proposed by Batista, using TDDFT calculations coupled with a continuum description of the environment and a micro-hydration scheme (Batista and Martin, 2005). Batista suggested the presence of two quasi-degenererate triplet states: one metal to ligand charge-transfer (MLCT) centered on the dppz (^3^MLCT_*dppz*_) and one intraligand (IL) ππ^*^ triplet still centered on the dppz (^3^IL_*dppz*_). The authors attribute the quenching of luminescence by water to the stabilization of the dark ^3^MLCT_*dppz*_ that becomes the lowest triplet state and hence easily populated. The stabilization would be mostly due to hydrogen bonds between solvent molecules and the nitrogen dppz atoms.

More recently a refined explanation for the light-switch effect was offered coupling a multiscale modeling to describe the interactions with the surroundings together with TDDFT and high level ab-initio calculations. Firstly the intercalation interaction mode and the main features of the absorption spectrum of [Ru(dppz)(bipy)_2_]^2+^ interacting with DNA were modeled, also taking into account polarizable embedding and validating the computational protocol (Very et al., 2012). To this end the presence of the charge-transfer band due to the Ruthenium complex was evidenced at around 450 nm. The band was assigned to a complex manifold of different metal-to-ligand (MLCT) transitions. Subsequently (Very et al., 2014), the triplet excited state manifold of [Ru(dppz)(bipy)_2_]^+2^ in different environments, namely water, acetophenone and DNA was computed, and the distribution of electronic density analyzed. The authors concluded that in water the dark ^3^MLCT_*dppz*_ state was populated being the lowest one upon geometry relaxation, in acetonitrile on the other hand the lowest triplet excited state was still of MLCT character but was centered both on the dppz and bipy ligands (^3^MLCT_*dppz*∕*bipy*_) since in contrast to the dppz centered one the ^3^MLCT_*bipy*_ are brights the mixed ^3^MLCT_*dppz*∕*bipy*_ can be considered as weakly emissive, coherently with the experiment. Finally in the case of intercalation in DNA the triplet state becomes now entirely centered on the bipy ancillary ligand (^3^MLCT_*bipy*_) giving rise to a bright state and hence to the triggering of luminescence. Therefore, the light-switch effect does not happen because of a competition between MLCT and IL triplet states but instead by a change in the electronic density reorganization of the MLCT states operated by the environment.

Usually photosensitization can be classified considering the physical phenomenon producing the lesion with DNA. Type I photosensitization is due to a charge transfer usually toward guanine, type II sensitization on the other hand is based on an energy-transfer between the sensitizer (triplet) excited state and molecular oxygen giving raise to ^1^O_2_ and the subsequent oxidative events. Finally, one should also take into account triplet photosensitization characterized by an energy-transfer between the photosensitizers and DNA bases, usually thymine; thymine dimers are the most common final products of triplet sensitization. Since the physical processes underlining these photosensitization pathways are different, modeling strategies should be tailored to provide a satisfactory description.

Concerning type I photosensitization some evidences of important electron transfer between nearby guanine and different ruthenium complexes have been reported already at Franck-Condon level using QM/MM performed with an extended QM partition and TDDFT calculations (Chantzis et al., 2013). Indeed, in this contribution the authors have calculated the absorption spectrum of ruthenium complexes intercalated in DNA extending the QM partition to accommodate the nearby guanine and adenine base. By analyzing the electron density reorganization, it has been shown that some vertical transitions exist around 300 nm characterized by a very strong charge-transfer, the calculation of natural population analysis (NPA) charges have allowed to estimate such charge-transfer to be close to 0.7 electron.

Also in the case of type II sensitization the role of the environment may be far from innocent. For instance in the case of a DNA interacting drug known as palmatine the production of ^1^O_2_ as well as fluorescence is triggered only by interaction with DNA (Hirakawa et al., 2012). Once again only QM/MM methods coupled with MD allows for a rationalization of the photochemical pathways (Dumont and Monari, 2015). Indeed, it was shown that in water solution upon excitation to a valence ππ^*^ (3.0 eV), state palmatine relaxation crosses a CT state that happens to be, at his relaxed geometry, lower in energy than the valence state (3.8 against 4.0 eV, respectively). This fact opens the way to efficient non-radiative pathways and hence induces quenching of luminescence and the inhibition of singlet oxygen production. In the case of the interactions with DNA (happening through minor-groove or insertion) already at Franck-Condon geometry the CT state is strongly destabilized by more than 1 eV comparing to the water-solution case. As a consequence the crossing with the ππ^*^ state is impossible even after geometry relaxation. Therefore, when interacting with DNA, palmatine evolves toward the S_1_ (ππ^*^) state that can subsequently either relax radiatively (fluorescence) or give intersystem crossing to populate the triplet state and produce ^1^O_2_. Intersystem crossing is also supported by the high spin-orbit coupling calculated to be of the order of 40 cm^−1^. We note that in this contribution (Dumont and Monari, 2015), it was evidenced that in the case of planar conjugated systems, the calculation of the absorption spectrum necessitates to take into account the effect of the low-frequency vibration modes, for instance through the coupling with MD. Indeed the calculation of absorption spectrum with static procedures gives totally unreliable results. Indeed, static absorption spectrum has a shift of about 100 nm compared to experimental results, while the inclusion of dynamic effects allows to perfectly match the observed absorption maximum (420 nm) providing one uses a long-range corrected functional. This situation was also observed for other DNA interacting systems such as the β-carboline harmane both for absorption and fluorescence spectra (Etienne et al., 2013, 2014).

The role of the environment in tuning type II photosensitization, and in particularly in driving the population of the triplet manifold was recently pointed out by Nogueira et al. in the case of the interaction between DNA and phenothiazinium dyes (Nogueira et al., 2015). The author evidenced that while in water solution intersystem-crossing is only driven by relatively inefficient vibronic spin-orbit coupling, the interaction with DNA modifies the energy order of the excited states and hence opens the way to a second symmetry allowed crossing. Indeed, in water solution the dye has to overcome significant barriers both from Franck-Condon region and S_1_ minimum (0.77 and 0.51 eV, respectively) to reach the symmetry allowed intersystem crossing between S_1_ and T_3_. On the other hand in DNA, mostly because of the hydrogen-bonding networks, the modified potential energy landscape for singlet and triplet results in a significant lowering of the barriers that assume values of 0.15 and 0.30 eV, for Franck-Condon or S_1_ minimum, respectively hence the intersystem crossing region can now be safely reached thanks to thermal energy and ^1^O_2_ ne produced. The observed production of singlet oxygen in water is ascribed to the coupling between electronic and vibrational movements, i.e., to the so called vibronic spin-orbit coupling, happening between S_1_ and T_2_ state, however this channel being less effective that the fully allowed electronic spin-orbit coupling with T_3_ the enhancement in the production of ^1^O_2_ by the DNA environment is confirmed.

Triplet photosensitization is also an experimentally-known component of DNA damage that can be timely tackled by modeling. Since it involves the preliminary population of the photosensitizer triplet state manifold, one may ask if the macromolecular environment keeps the road to photosensitization open, or if it hinders its development. Acetophenone is a known triplet photosensitizer (Epe, 2012) and its efficiency is due to a very efficient population of the triplet state in gas phase (Huix-Rotllant et al., 2013). Molecular dynamics simulations have allowed to identify acetophenone stable interaction mode with DNA, namely intercalation (Huix-Rotllant et al., 2015). Furthermore, QM/MM calculations, performed both at CAS-PT2 and TDDFT level of theory, have shown that the gas-phase quasi-degeneracy of the first singlet excited state S_1_ with the two ππ^*^ and nπ^*^ triplet state is not significantly altered by the environment (Huix-Rotllant et al., 2015). The authors have also evidenced that all along the MD trajectory the spin-orbit coupling between the S_1_ and at least one of the triplet is always quite high (about 20 cm^−1^), i.e., a value that allows efficient intersystem crossing if the energy difference between the states is small (Richter et al., 2012). One of the most striking features of gas phase acetophenone photohysics, explaining the high-efficiency of the intersystem crossing is indeed the presence of a three-point crossing between the singlet and the two triplets. In presence of DNA although the perfect crossing is lifted the two intersections still happens in a very close-by region of the configuration space (Huix-Rotllant et al., 2015). All these results point toward the fact that the DNA environment does not alter the efficient photophysical pathways leading to acetophenone triplet population during the very first vibrational motions. This in turn points toward the rationalization of the efficient DNA photosensitization experienced by acetophenone.

But in order to be efficient in terms of triplet-photosensitization, the population of the triplet manifold should be rapidly followed by energy transfer to the nearby thymine. This aspect has been recently deeply analyzed for the case of benzophenone (Dumont et al., 2015). The energy profile for the benzophenone-DNA triplet transfer has been obtained following an approximate reaction coordinate both for a double-inserted and a minor-groove bound benzophenone. Calculations have been performed by QM/MM to take into account the environment effects, while triplet energy has been obtained both at CASPT2 and TD-DFT level. In particular it has been evidenced that for double-insertion two very efficient and barrierless sensitization channels exist one proceeding from the T_1_ state (nπ^*^) and the other from the T_2_ (ππ^*^) triplet. On the contrary in the case of minor-groove binding the T_1_ sensitization channel is hampered by a significant barrier (0.30 eV), while the T_2_ channel is still active and virtually barrierless. Furthermore, it has been evidenced that due to the close-by distance of the interacting chromophores the transfer will proceed through the Dexter mechanism mainly driven by the electronic densities overlap. Estimation of the overlap has shown a slightly pronounced kinetic preference for the double-insertion mode, but still shows that the transfer from minor-groove is possible and efficient although with a 100 times lower efficiency compared to the double-inserted situation.

Obviously a deeper characterization of energy- and electron-transfer processes will necessitate to take into account non-adiabatic and state-hopping dynamics. Furthermore, since the triplet manifolds are involved in most of the cases the spin-orbit coupling should not be neglected. This aspect coupled with the necessity to take into account the environment and many interacting chromophores and the relative long time scale of the full sensitization process will necessitate a considerable computational effort that can be achieved only through a proper methodological development. Still significant progresses have been made allowing to get a clearer vision of a fundamental and rather complicated process whose biological relevance cannot be underestimated.

## 4. Sensitizing non-canonical DNA structures

Even if the canonical B-DNA form is by far the most common one, particular DNA sequences may adopt the so-called non-canonical conformations. The biological relevance of such structures is now clearly evidenced. On that context particular significance has to be attributed to the guanine-quadruplex (G4), that are present in guanine-rich sequences such as the telomeres (Neidle and Parkinson, 2003). Telomeres protecting activity is a key factor to regulate cellular apoptosis, indeed the activation of telomerase, which is overexpressed in a vast majority of tumor cell lines, is strongly related to cancer cells uncontrolled replication (Neidle and Parkinson, 2002). The stabilization of G4 structures by DNA sensitization may result in an inhibition of the telomerase (Neidle, 2010), hence opening the way to novel anticancer therapies. In particular the selectivity of the sensitizer toward G4, instead of canonical DNA structure, will be a key factor governing the overall selectivity of the drug toward cancer cells.

The relative peculiar structure of G4 has necessitated an important computational effort to rationalize their structure and their stability (Ilchenko and Dubey, 2014), as well as the the sequence effects governing their formation. Furthermore, since G4 are stabilized by interactions with cations to compensate the negative charges the pH and salt concentrations are key factors determining their appearance. In a vast majority of studies (Ilchenko and Dubey, 2014) QM methods were used to analyze the stabilizing factors of G4. Systematic DFT studies were performed (Jissy et al., 2011), in addition the role of different cations stabilizing the central channel was investigated in particularly connected to the emergence of polarizability anisotropy that may induce birifrangence potentially exploitable in biomolecular imaging.

A rather serious complication in the study of G4 is connected to the polymorphism observed for such structures (Ilchenko and Dubey, 2014). This experimentally observed evidence, has also been rationalized by other DFT calculations (Fonseca Guerra et al., 2010), in particular the role of the hydrogen-bond networks has been evidenced. Some of the same authors have also proposed new possible monomers leading to the formation of quadruplexes structure such as substituted xanthines (Szolomajer et al., 2011).

In parallel, the necessity to correctly represent the polymorphism of the G4 non-canonical structures has pushed toward the validation of force-fields, which are usually parameterized to reproduce the behavior of canonical B-DNA (Grunenberg et al., 2014). The comparison of force field descriptions with DFT calculations shows that extreme care should be taken to model the competitive rotamers. In particular while the OPLS and the MMFF force fields correctly reproduce experimental and DFT results, the DNA-popular Amber force field gives the opposite energy-order.

However, the combination of well parameterized force fields and of QM and QM/MM techniques allows to correctly reproduce the interaction of sensitizers with G4. The most common G4 sensitizers are rather extended and planar π-conjugated cations (Ilchenko and Dubey, 2014). Recently, Barone group (Terenzi et al., 2014) has used molecular modeling to predict the relative stability of G4 vs. B-DNA aggregates with square-planar organometallic Schiff-base complexes containing copper and zinc. MD simulations have shown that the Schiff-base strongly interact with G4 both via electrostatic interactions and through efficient π-stacking. The estimation of the G4/sensitizer free energy of formation gives values ranging from −34.6 to −14.4 kcal/mol therefore speaking in favor of a stable complex. It is noteworthy that these results have also been supported by biological tests confirming a potential anti-cancer activity.

It is evident that despite some difficulty the sensitization of non-canonical DNA structure, both at the ground and for the excited-state, is no more considered as an exotic novelty. On the contrary it is displaying all its potentiality in the rational design of novel therapeutic strategies. Molecular modeling is playing an active role on the field and will certainly drive not only the comprehension of the mechanisms leading to a selective sensitization, but also the prediction of entirely novel sensitizers.

## 5. Conclusions

The impressive progresses of modeling and simulation techniques have now made possible tackling problems as complex as the generation of oxidatively-induced DNA lesions and the interaction of DNA with potential drugs. However, this domain should still be considered very challenging also from a computational chemistry point of view. Indeed, many different factors should be taken into account and treated on the same footing to have a proper, and biologically relevant, description of the various phenomena comming into play.

Particular attention should be taken for instance to model the conformational degrees of freedom of the very flexible DNA macromolecule, this of course calls for adequate and long-scale molecular dynamics trajectory. Techniques allowing a good estimate of the free-energy, especially but not exclusively, in the case of sensitization binding energies are also of paramount importance. This aspect will of course require the development of efficient force fields as well as of proper sampling techniques.

The study of the electron density reorganization taking place on the ground- or on the excited states is also fundamental to go beyond the simple structural description and propose hints on the reactivity or the photochemistry of DNA alone or in presence of sensitizers. The envisaged QM or QM/MM techniques should conjugate the precision of the description with the efficiency necessary to make possible the sampling of important region of the configuration space, and hence having access to good statistical properties. This is especially true since in many case energy- or electron-transfer phenomena have to be taken into due accounts, i.e., one has to deal with some of the most challenging situations for QM methods.

We believe that from a methodological point of view the development of novel multiscale techniques in particular for what concerns excited state non-adiabatic dynamics as well as ab-initio QM/MM dynamics will allow the treatment of more and more complex scenarios, coupling a good accuracy with good statistics. On that context the development of proper sampling techniques allowing the definition of good and physical relevant reaction coordinates taking into account all the relevant collective variables will certainly be one of the most important challenges of the next years.

Nowadays, the atomistic scale description of DNA related phenomena offered by multiscale modeling allows to rationalize and properly describe phenomena as diverse as the behavior of DNA under oxidative stress or the environment controlled behavior of phototherapy drugs. The selectivity of potential drug candidates toward different DNA configurations as well as the differential dynamical behavior of different DNA sequences are now accessible at least at a semi-quantitative level.

Modern day molecular modeling is therefore able to provide a molecular and electronic description of the factor governing DNA chemistry under external perturbation, therefore molecular modeling can nowadays be considered as a fundamental tool to provide unprecedented insights in chemical biology. Conversely, the knowledge of the mechanism of DNA lesions induction are also paving the way to the rational design of novel therapeutic agents. Therefore we may safely say that the communication between the *in silico* world of molecular modeling and the *in vitro* world of biological chemistry and cellular biology is now a sounding and promising reality.

## Author contributions

Both authors contributed equally to the present review.

### Conflict of interest statement

The authors declare that the research was conducted in the absence of any commercial or financial relationships that could be construed as a potential conflict of interest.

## References

[B1] Abolfath R. M. Biswas P. K. Rajnarayanam R. Brabec T. Kodym R. Papiez L. (2012). Multiscale QM/MM molecular dynamics study on the first steps of guanine damage by free hydroxyl radicals in solution. J. Phys. Chem. A 116, 3940–3945. 10.1021/jp300258n 22397677 PMC3356683

[B2] Abolfath R. M. van Duin A. C. T. Brabec T. (2011). Reactive molecular dynamics study on the first steps of DNA damage by free hydroxyl radicals. J. Phys. Chem. A 115, 11045–11049. 10.1021/jp204894m 21882859

[B3] Agostinis P. Berg K. Cengel K. A. Foster T. H. Girotti A. W. Gollnick S. O. (2011). Photodynamic therapy of cancer: an update. Cancer J. Clin. 61, 250–281. 10.3322/caac.20114 PMC3209659 21617154

[B4] Antonarakis E. S. Emadi A. (2010). Ruthenium-based chemotherapeutics: are they ready for prime time? Cancer Chemother. Pharmacol. 66, 1–9. 10.1007/s00280-010-1293-1 20213076 PMC4020437

[B5] Balasubramanian B. Pogozelski W. K. Tullius T. D. (1998). DNA strand breaking by the hydroxyl radical is governed by the accessible surface areas of the hydrogen atoms of the DNA backbone. Proc. Natl. Acad. Sci. U.S.A. 95, 9738–9743. 10.1073/pnas.95.17.9738 9707545 PMC21406

[B6] Banyasz A. Douki T. Improta R. Gustavsson T. Onidas D. Vayá I.. (2012). Electronic excited states responsible for dimer formation upon UV absorption directly by thymine strands: joint experimental and theoretical study. J. Am. Chem. Soc. 134, 14834–14845. 10.1021/ja304069f 22894169

[B7] Barbatti M. (2011). Nonadiabatic dynamics with trajectory surface hopping method. Wiley Interdiscipl. Rev. Comput. Mol. Sci. 1, 620–633. 10.1002/wcms.64

[B8] Barbatti M. Aquino A. J. A. Szymczak J. J. Nachtigallová D. Hobza P. Lischka H. (2010). Relaxation mechanisms of UV-photoexcited DNA and RNA nucleobases. Proc. Natl. Acad. Sci. U.S.A. 107, 21453–21458. 10.1073/pnas.1014982107 21115845 PMC3003128

[B9] Barnett R. N. Bongiorno A. Cleveland C. L. Joy A. Landman U. Schuster G. B. (2006). Oxidative damage to DNA: counterion-assisted addition of water to ionized dna. J. Am. Chem. Soc. 128, 10795–10800. 10.1021/ja061795y 16910674

[B10] Batista E. R. Martin R. L. (2005). On the excited states involved in the luminescent probe [Ru(bpy)2dppz]2+. J. Phys. Chem. A 109, 3128–3133. 10.1021/jp050673+ 16833639

[B11] Bera P. P. Schaefer H. F. (2005). (GH)^•^C and G(CH)^•^ radicals derived from the guanine–cytosine base pair cause dna subunit lesions. Proc. Natl. Acad. Sci. U.S.A. 102, 6698–6703. 10.1073/pnas.0408644102 15814617 PMC1100756

[B12] Bergeron F. Auvre F. Radicella J. P. Ravanat J.-L. (2010). HO· radicals induce an unexpected high proportion of tandem base lesions refractory to repair by DNA glycosylases. Proc. Natl. Acad. Sci. U.S.A. 107, 5528–5533. 10.1073/pnas.1000193107 20212167 PMC2851781

[B13] Besic E. Sankovic K. Gomzi V. Herak J. N. (2001). Sigma radicals in gamma-irradiated single crystals of 2-thiothymine. Phys. Chem. Chem. Phys. 3, 2723–2725. 10.1039/b103210k

[B14] Brabec V. Reedijk J. Leng M. (1992). Sequence-dependent distortions induced in dna by monofunctional platinum(II) binding. Biochemistry 31, 12397–12402. 10.1021/bi00164a014 1463726

[B15] Brash D. E. (2015). UV signature mutations. Photochem. Photobiol. 91, 15–26. 10.1111/php.12377 25354245 PMC4294947

[B16] Bulent A. Wesley E. B. Steven G. S. James E. T. David T. M.. (2008). (monte carlo) simulations of site-specific radical attack to DNA bases. Radiat. Res. 169, 223–231. 10.1667/RR0293.1 18220458

[B17] Cadenas E. Davies K. J. (2000). Mitochondrial free radical generation, oxidative stress, and aging1. Free Rad. Biol. Med. 29, 222–230. 10.1016/S0891-5849(00)00317-8 11035250

[B18] Cadet J. Delatour T. Douki T. Gasparutto D. Pouget J.-P. Ravanat J.-L. (1999). Hydroxyl radicals and DNA base damage. Mutat. Res. Fundament. Mol. Mech. Mutagenes. 424, 9–21. 10.1016/S0027-5107(99)00004-4 10064846

[B19] Cadet J. Wagner J. R. (2013). DNA base damage by reactive oxygen species, oxidizing agents, and UV radiation. Cold Spring Harb. Perspect. Biol. 5:a012559. 10.1101/cshperspect.a012559 23378590 PMC3552502

[B20] Carvlin M. J. Datta-Gupta N. Fiel R. J. (1982). Circular dichroism spectroscopy of a cationic porphyrin bound to DNA. Biochem. Biophys. Res. Commun. 108, 66–73. 10.1016/0006-291X(82)91832-0 7150298

[B21] Cauët E. Bogatko S. Lievin J. De Proft F. Geerlings P. (2013). Electron-attachment-induced DNA damage: instantaneous strand breaks. J. Phys. Chem. B 117, 9669–9676. 10.1021/jp406320g 23869464

[B22] Ceron-Carrasco J. P. Jacquemin D. (2013). Electric field induced DNA damage: an open door for selective mutations. Chem. Commun. 49, 7578–7580. 10.1039/c3cc42593b 23722648

[B23] Chaiyen P. Fraaije M. W. Mattevi A. (2012). The enigmatic reaction of flavins with oxygen. Trends Biochem. Sci. 37, 373–380. 10.1016/j.tibs.2012.06.005 22819837

[B24] Chantzis A. Very T. Daniel C. Monari A. Assfeld X. (2013). Theoretical evidence of photo-induced charge transfer from DNA to intercalated ruthenium (II) organometallic complexes. Chem. Phys. Lett. 578, 133–137. 10.1016/j.cplett.2013.05.068

[B25] Chen J. Dupradeau F.-Y. Case D. A. Turner C. J. Stubbe J. (2008). DNA oligonucleotides with A, T, G or C opposite an abasic site: structure and dynamics. Nucleic Acids Res. 36, 253–262. 10.1093/nar/gkm622 18025040 PMC2248740

[B26] Cooke M. S. Evans M. D. Dizdaroglu M. Lunec J. (2003). Oxidative DNA damage: mechanisms, mutation, and disease. FASEB J. 17, 1195–1214. 10.1096/fj.02-0752rev 12832285

[B27] Cuquerella M. C. Lhiaubet-Vallet V. Cadet J. Miranda M. A. (2012). Benzophenone photosensitized dna damage. Accoun. Chem. Res. 45, 1558–1570. 10.1021/ar300054e 22698517

[B28] Ding S. Kolbanovskiy A. Durandin A. Crean C. Shafirovich V. Broyde S.. (2009). Absolute configurations of DNA lesions determined by comparisons of experimental ECD and ORD spectra with DFT calculations. Chirality 21, E231–E241. 10.1002/chir.20804 19937959 PMC4033673

[B29] Dougherty T. J. Gomer C. J. Henderson B. W. Jori G. Kessel D. Korbelik M.. (1998). Photodynamic therapy. J. Natl. Cancer Inst. 90, 889–905. 10.1093/jnci/90.12.889 9637138 PMC4592754

[B30] Dumont E. Monari A. (2013). Benzophenone and DNA: evidence for a double insertion mode and its spectral signature. J. Phys. Chem. Lett. 4, 4119–4124. 10.1021/jz4021475

[B31] Dumont E. Monari A. (2015). Interaction of palmatine with DNA: an environmentally controlled phototherapy drug. J. Phys. Chem. B 119, 410–419. 10.1021/jp5088515 25526561

[B32] Dumont E. Wibowo M. Roca-Sanjuan D. Garavelli M. Assfeld X. Monari A. (2015). Resolving the benzophenone DNA-photosensitization mechanism at QM/MM level. J. Phys. Chem. Lett. 6, 576–580. 10.1021/jz502562d 26262469

[B33] Duncan Lyngdoh R. H. Schaefer H. F. (2009). Elementary lesions in DNA subunits: electron, hydrogen atom, proton, and hydride transfers. Acc. Chem. Res. 42, 563–572. 10.1021/ar800077q 19231845

[B34] Durbeej B. Eriksson L. A. (2003). On the formation of cyclobutane pyrimidine dimers in UV-irradiated DNA: why are thymines more reactive? Photochem. Photobiol. 78, 159–167. 10.1562/0031-8655(2003)078<0159:OTFOCP>2.0.CO;2 12945584

[B35] Epe B. (2012). DNA damage spectra induced by photosensitization. Photochem. Photobiol. Sci. 11, 98–106. 10.1039/C1PP05190C 21901212

[B36] Esposito L. Banyasz A. Douki T. Perron M. Markovitsi D. Improta R. (2014). Effect of c5-methylation of cytosine on the photoreactivity of DNA: a joint experimental and computational study of TCG trinucleotides. J. Am. Chem. Soc. 136, 10838–10841. 10.1021/ja5040478 25050452

[B37] Ethirajan M. Chen Y. Joshi P. Pandey R. K. (2011). The role of porphyrin chemistry in tumor imaging and photodynamic therapy. Chem. Soc. Rev. 40, 340–362. 10.1039/B915149B 20694259

[B38] Etienne T. Gattuso H. Monari A. Assfeld X. (2014). QM/MM modeling of Harmane cation fluorescence spectrum in water solution and interacting with DNA. Comput. Theor. Chem. 1040–1041, 367–372. 10.1016/j.comptc.2014.03.026

[B39] Etienne T. Very T. Perpète E. A. Monari A. Assfeld X. (2013). A QM/MM study of the absorption spectrum of harmane in water solution and interacting with DNA: the crucial role of dynamic effects. J. Phys. Chem. B 117, 4973–4980. 10.1021/jp4017882 23541279

[B40] Finkel T. (2011). Signal transduction by reactive oxygen species. J. Cell Biol. 194, 7–15. 10.1083/jcb.201102095 21746850 PMC3135394

[B41] Florea A.-M. Büsselberg D. (2011). Cisplatin as an anti-tumor drug: cellular mechanisms of activity, drug resistance and induced side effects. Cancers 3, 1351–1371. 10.3390/cancers3011351 24212665 PMC3756417

[B42] Fonseca Guerra C. van der Wijst T. Poater J. Swart M. Bickelhaupt F. (2010). Adenine versus guanine quartets in aqueous solution: dispersion-corrected DFT study on the differences in π-stacking and hydrogen-bonding behavior. Theor. Chem. Acc. 125, 245–252. 10.1007/s00214-009-0634-9

[B43] Frances-Monneris A. Merchan M. Roca-Sanjuan D. (2013). Electronic UV-Vis transient spectra of the oh reaction products of uracil, thymine, cytosine, and 5,6-dihydrouracil by using the complete active space self-consistent field second-order perturbation (CASPT2//CASSCF) theory. J. Chem. Phys. 139:071101. 10.1063/1.4818727 23968062

[B44] Frelon S. Douki T. Ravanat J.-L. Pouget J.-P. Tornabene C. Cadet J. (2000). High-performance liquid chromatographytandem mass spectrometry measurement of radiation-induced base damage to isolated and cellular DNA. Chem. Res. Toxicol. 13, 1002–1010. 10.1021/tx000085h 11080049

[B45] Fridovich I. (1995). Superoxide radical and superoxide dismutases. Annu. Rev. Biochem. 64, 97–112. 10.1146/annurev.bi.64.070195.000525 7574505

[B46] Friedman A. E. Chambron J. C. Sauvage J. P. Turro N. J. Barton J. K. (1990). A molecular light switch for DNA: Ru(bpy)2(dppz)2+. J. Am. Chem. Soc. 112, 4960–4962. 10.1021/ja00168a052

[B47] Galindo-Murillo R. Garcia-Ramos J. C. Ruiz-Azuara L. Cheatham T. E. Cortes-Guzman F. (2015). Intercalation processes of copper complexes in DNA. Nucleic Acids Res. 10.1093/nar/gkv467 PMC4477671 25958394

[B48] Garcia-Mesenguer R. Marti S. Ruiz-Pernia J. J. Moliner V. Tunon I. (2013). Studying the role of protein dynamics in an SN2 enzyme reaction using free-energy surfaces and solvent coordinates. Nat. Chem. 5, 566–571. 10.1038/nchem.1660 23787745

[B49] Garrec J. Patel C. Rothlisberger U. Dumont E. (2012). Insights into intrastrand cross-link lesions of DNA from QM/MM molecular dynamics simulations. J. Am. Chem. Soc. 134, 2111–2119. 10.1021/ja2084042 22200321

[B50] Gervasio F. L. Laio A. Iannuzzi M. Parrinello M. (2004). Influence of DNA structure on the reactivity of the guanine radical cation. Chem. Eur. J. 10, 4846–4852. 10.1002/chem.200400171 15372666

[B51] Gold B. Stone M. P. Marky L. A. (2014). Looking for Waldo: a potential thermodynamic signature to DNA damage. Acc. Chem. Res. 47, 1446–1454. 10.1021/ar500061p 24702131 PMC3993888

[B52] Gomzi V. (2011). {DFT} study of radicals formed in 2-thiothymine single crystals at 77k: 1- and 2-molecule models revised. Comput. Theor. Chem. 963, 497–502. 10.1016/j.comptc.2010.11.019

[B53] Groenhof G. Schäfer L. V. Boggio-Pasqua M. Goette M. Grubmüller H. Robb M. A. (2007). Ultrafast deactivation of an excited cytosineguanine base pair in DNA. J. Am. Chem. Soc. 129, 6812–6819. 10.1021/ja069176c 17488008

[B54] Grunenberg J. Barone G. Spinello A. (2014). The right answer for the right electrostatics: force field methods are able to describe relative energies of DNA guanine quadruplexes. J. Chem. Theory Comput. 10, 2901–2905. 10.1021/ct500329f 26588265

[B55] Gustavsson T. Improta R. Markovitsi D. (2010). DNA/RNA: building blocks of life under UV irradiation. J. Phys. Chem. Lett. 1, 2025–2030. 10.1021/jz1004973

[B56] Gustavsson T. Sarkar N. Vaya I. Jimenez M. C. Markovitsi D. Improta R. (2013). A joint experimental/theoretical study of the ultrafast excited state deactivation of deoxyadenosine and 9-methyladenine in water and acetonitrile. Photochem. Photobiol. Sci. 12, 1375–1386. 10.1039/c3pp50060h 23615844

[B57] Hamanaka R. B. Chandel N. S. (2010). Mitochondrial reactive oxygen species regulate cellular signaling and dictate biological outcomes. Trends Biochem. Sci. 35, 505–513. 10.1016/j.tibs.2010.04.002 20430626 PMC2933303

[B58] Hirakawa K. Hirano T. Nishimura Y. Arai T. Nosaka Y. (2012). Dynamics of singlet oxygen generation by DNA-binding photosensitizers. J. Phys. Chem. B 116, 3037–3044. 10.1021/jp300142e 22313410

[B59] Huix-Rotllant M. Dumont E. Ferré N. Monari A. (2015). Photophysics of acetophenone interacting with DNA: why the road to photosensitization is open. Photochem. Photobiol. 91, 323–330. 10.1111/php.12395 25412588

[B60] Huix-Rotllant M. Siri D. Ferré N. (2013). Theoretical study of the photochemical generation of triplet acetophenone. Phys. Chem. Chem. Phys. 15, 19293–19300. 10.1039/c3cp52703d 24113419

[B61] Hutter M. Clark T. (1996). On the enhanced stability of the guaninecytosine base-pair radical cation. J. Am. Chem. Soc. 118, 7574–7577. 10.1021/ja953370+

[B62] Ilchenko M. Dubey I. (2014). Quantum chemical approaches in modeling the structure of DNA quadruplexes and their interaction with metal ions and small molecules, in Application of Computational Techniques in Pharmacy and Medicine, Volume 17 of *Challenges and Advances in Computational Chemistry and Physics*, eds Gorb L. Kuz'min V. Muratov E. (Berlin: Springer), 181–206.

[B63] Jacquemin D. Zuniga J. Requena A. Ceron-Carrasco J. P. (2014). Assessing the importance of proton transfer reactions in DNA. Acc. Chem. Res. 47, 2467–2474. 10.1021/ar500148c 24849375

[B64] Jain V. Vaidyanathan V. G. Patnaik S. Gopal S. Cho B. P. (2014). Conformational insights into the lesion and sequence effects for arylamine-induced translesion DNA synthesis: 19F NMR, surface plasmon resonance, and primer kinetic studies. Biochemistry 53, 4059–4071. 10.1021/bi5003212 24915610 PMC4075988

[B65] Ji Y. Xia Y. Zhao M. Li F. Huang B. (2005). Reactions of ·OH with thymine studied using density functional theory. Int. J. Quant. Chem. 101, 211–218. 10.1002/qua.20293

[B66] Jissy A. Ashik U. Datta A. (2011). Nucleic acid G-quartets: insights into diverse patterns and optical properties. J. Phys. Chem. C 115, 12530–12546. 10.1021/jp202401b

[B67] Johnson E. R. Keinan S. Mori-Sánchez P. Contreras-García J. Cohen A. J. Yang W. (2010). Revealing noncovalent interactions. J. Am. Chem. Soc. 132, 6498–6506. 10.1021/ja100936w 20394428 PMC2864795

[B68] Jurecka P. Sponer J. Cerny J. Hobza P. (2006). Benchmark database of accurate (MP2 and CCSD(T) complete basis set limit) interaction energies of small model complexes, DNA base pairs, and amino acid pairs. Phys. Chem. Chem. Phys. 8, 1985–1993. 10.1039/b600027d 16633685

[B69] Kamp D. W. Shacter E. Weitzman S. A. (2011). Chronic inflammation and cancer: the role of the mitochondria. Oncology 25, 400–410, 413. 21710835

[B70] Kielpinski L. J. Vinther J. (2014). Massive parallel-sequencing-based hydroxyl radical probing of RNA accessibility. Nucleic Acids Res. 42:e70. 10.1093/nar/gku167 24569351 PMC4005689

[B71] Klaunig J. E. Kamendulis L. M. Hocevar B. A. (2010). Oxidative stress and oxidative damage in carcinogenesis. Toxicol. Pathol. 38, 96–109. 10.1177/0192623309356453 20019356

[B72] Kujoth G. C. Hiona A. Pugh T. D. Someya S. Panzer K. Wohlgemuth S. E.. (2005). Mitochondrial DNA mutations, oxidative stress, and apoptosis in mammalian aging. Science 309, 481–484. 10.1126/science.1112125 16020738

[B73] Labet V. Morell C. Grand A. Cadet J. Cimino P. Barone V. (2008). Formation of cross-linked adducts between guanine and thymine mediated by hydroxyl radical and one-electron oxidation: a theoretical study. Organ. Biomol. Chem. 6, 3300–3305. 10.1039/b805589k 18802636

[B74] Labet V. Morell C. Tognetti V. Syzgantseva O. A. Joubert L. Jorge N. (2014). Characterization of the chemical reactivity and selectivity of DNA bases through the use of DFT-based descriptors, in Structure, Bonding and Reactivity of Heterocyclic Compounds, Volume 38 of *Topics in Heterocyclic Chemistry*, eds De Proft F. Geerlings P. (Berlin; Heidelberg: Springer), 35–70.

[B75] Lauria A. Bonsignore R. Terenzi A. Spinello A. Giannici F. Longo A.. (2014). Nickel(ii), copper(ii) and zinc(ii) metallo-intercalators: structural details of the DNA-binding by a combined experimental and computational investigation. Dalton Trans. 43, 6108–6119. 10.1039/c3dt53066c 24522514

[B76] Lim P. Wuenschell G. E. Holland V. Lee D.-H. Pfeifer G. P. Rodriguez H.. (2004). Peroxyl radical mediated oxidative DNA base damage: implications for lipid peroxidation induced mutagenesis. Biochemistry 43, 15339–15348. 10.1021/bi048276x 15581346

[B77] Meier K. Choutko A. Dolenc J. Eichenberger A. P. Riniker S. van Gunsteren W. F. (2013). Multi-resolution simulation of biomolecular systems: a review of methodological issues. Angewandte Chemie Int. Edn. 52, 2820–2834. 10.1002/anie.201205408 23417997

[B78] Miyamoto S. Di Mascio P. (2014). Lipid hydroperoxides as a source of singlet molecular oxygen, in Lipid Hydroperoxide-Derived Modification of Biomolecules, Volume 77 of *Subcellular Biochemistry*, ed Kato Y. (Berlin: Springer), 3–20. 10.1007/978-94-007-7920-4_1 24374914

[B79] Monari A. Rivail J.-L. Assfeld X. (2013). Theoretical modeling of large molecular systems. advances in the local self consistent field method for mixed quantum mechanics/molecular mechanics calculations. Acc. Chem. Res. 46, 596–603. 10.1021/ar300278j 23249409

[B80] Mutter S. T. Margiotta N. Papadia P. Platts J. A. (2015). Computational evidence for structural consequences of kiteplatin damage on DNA. JBIC J. Biol. Inorgan. Chem. 20, 35–48. 10.1007/s00775-014-1207-5 25377895

[B81] Neidle S. (2010). Human telomeric G-quadruplex: the current status of telomeric G-quadruplexes as therapeutic targets in human cancer. FEBS J. 277, 1118–1125. 10.1111/j.1742-4658.2009.07463.x 19951354

[B82] Neidle S. Parkinson G. (2002). Telomere maintenance as a target for anticancer drug discovery. Nat. Rev. Drug Dis. 1, 383–393. 10.1038/nrd793 12120414

[B83] Neidle S. Parkinson G. N. (2003). The structure of telomeric DNA. Curr. Opin. Struct. Biol. 13, 275–283. 10.1016/S0959-440X(03)00072-1 12831878

[B84] Nenov A. Segarra-Marti J. Giussani A. Conti I. Rivalta I. Dumont E.. (2015). Probing deactivation pathways of DNA nucleobases by two-dimensional electronic spectroscopy: first principles simulations. Faraday Discuss. 177, 345–362. 10.1039/C4FD00175C 25607949

[B85] Nielsen S. O. Bulo R. E. Moore P. B. Ensing B. (2010). Recent progress in adaptive multiscale molecular dynamics simulations of soft matter. Phys. Chem. Chem. Phys. 12, 12401–12414. 10.1039/c004111d 20734007

[B86] Nijkoo H. O'Neill P. M. T. Goodhead D. T. (1999). Quantitative modelling of DNA damage using Monte Carlo track structure method. Radiat. Environ. Biophys. 38, 31–38. 10384953 10.1007/s004110050135

[B87] Nikitaki Z. Hellweg C. E. Georgakilas A. G. Ravanat J.-L. (2015). Stress-induced DNA damage biomarkers: applications and limitations. Front. Chem. 3:35. 10.3389/fchem.2015.00035 26082923 PMC4451417

[B88] Nogueira J. J. Oppel M. Gonzlez L. (2015). Enhancing intersystem crossing in phenotiazinium dyes by intercalation into DNA. Angewandte Chemie Int. Edn. 54, 4375–4378. 10.1002/anie.201411456 25663283

[B89] Pacher P. Beckman J. S. Liaudet L. (2007). Nitric oxide and peroxynitrite in health and disease. Physiol. Rev. 87, 315–424. 10.1152/physrev.00029.2006 17237348 PMC2248324

[B90] Pandey R. K. (2000). Recent advances in photodynamic therapy. J. Porphyr. Phthalocyan. 4, 368–373. 10.1002/(SICI)1099-1409(200006/07)4:4<368::AID-JPP244>3.0.CO;2-6

[B91] Patel C. Drsata T. Lankas F. Dumont E. (2013). Structure, dynamics, and interactions of a C4'-oxidized abasic site in DNA: a concomitant strand scission reverses affinities. Biochemistry 52, 8115–8125. 10.1021/bi401268q 24131173

[B92] Poater J. Swart M. Guerra C. F. Bickelhaupt F. M. (2011). Selectivity in DNA replication. interplay of steric shape, hydrogen bonds, π-stacking and solvent effects. Chem. Commun. 47, 7326–7328. 10.1039/c0cc04707d 21611661

[B93] Pogozelski W. K. Tullius T. D. (1998). Oxidative strand scission of nucleic acids: routes initiated by hydrogen abstraction from the sugar moiety. Chem. Rev. 98, 1089–1108. 10.1021/cr960437i 11848926

[B94] Pryor W. A. Squadrito G. L. (1995). The chemistry of peroxynitrite: a product from the reaction of nitric oxide with superoxide. Am. J. Physiol. Lung Cell. Mol. Physiol. 268, L699–L722. 10.1152/ajplung.1995.268.5.L699 7762673

[B95] Rademaker-Lakhai J. M. van den Bongard D. Pluim D. Beijnen J. H. Schellens J. H. M. (2004). A phase I and pharmacological study with imidazolium-trans-DMSO-imidazole-tetrachlororuthenate, a novel ruthenium anticancer agent. Clin. Cancer Res. 10, 3717–3727. 10.1158/1078-0432.CCR-03-0746 15173078

[B96] Radzimanowski J. Dehez F. Round A. Bidon-Chanal A. McSweeney S. Timmins J. (2013). An 'open' structure of the RecOR complex supports ssDNA binding within the core of the complex. Nucleic Acids Res. 7972–7986. 10.1093/nar/gkt572 23814185 PMC3763555

[B97] Ramsay R. R. (2012). Monoamine oxidases: the biochemistry of the proteins as targets in medicinal chemistry and drug discovery. Curr. Topics Med. Chem. 12, 2189–2209. 10.2174/156802612805219978 23231396

[B98] Regulus P. Duroux B. Bayle P.-A. Favier A. Cadet J. Ravanat J.-L. (2007). Oxidation of the sugar moiety of DNA by ionizing radiation or bleomycin could induce the formation of a cluster DNA lesion. Proc. Natl. Acad. Sci. U.S.A. 104, 14032–14037. 10.1073/pnas.0706044104 17715301 PMC1955805

[B99] Repic M. Vianello R. Purg M. Duarte F. Bauer P. Kamerlin S. C. L. (2014). Empirical valence bond simulations of the hydride transfer step in the monoamine oxidase B catalyzed metabolism of dopamine. Proteins Struct. Funct. Bioinform. 82, 3347–3355. 10.1002/prot.24690 25220264

[B100] Richter M. Marquetand P. Gonzĺez-Vázquez J. Sola I. González L. (2012). Femtosecond intersystem crossing in the DNA nucleobase cytosine. J. Phys. Chem. Lett. 3, 3090–3095. 10.1021/jz301312h 26296011

[B101] Sage E. Drouin R. Rouabhia M. (eds.). (2005). Chemical sequencing profiles of photosensitized DNA damage, in From DNA Photolesions to Mutations, Skin Cancer and Cell Death (Cambridge, UK: The Royal Society of Chemistry), 15–31.

[B102] Sainz R. M. Lombo F. Mayo J. C. (2012). Radical decisions in cancer: redox control of cell growth and death. Cancers 4, 442–474. 10.3390/cancers4020442 24213319 PMC3712695

[B103] Salmon T. B. Evert B. A. Song B. Doetsch P. W. (2004). Biological consequences of oxidative stress-induced DNA damage in saccharomyces cerevisiae. Nucleic Acids Res. 32, 3712–3723. 10.1093/nar/gkh696 15254273 PMC484183

[B104] Sancar A. Sancar G. B. (1988). DNA repair enzymes. Annu. Rev. Biochem. 57, 29–67. 10.1146/annurev.bi.57.070188.000333 3052275

[B105] Senn H. Thiel W. (2007). QM/MM methods for biological systems, in Atomistic Approaches in Modern Biology, Volume 268 of *Topics in Current Chemistry*, ed Reiher M. (Berlin; Heidelberg: Springer), 173–290.

[B106] Senn H. M. Thiel W. (2009). QM/MM methods for biomolecular systems. Angewandte Chemie Int. Edn. 48, 1198–1229. 10.1002/anie.200802019 19173328

[B107] Sinha R. P. Hader D.-P. (2002). UV-induced DNA damage and repair: a review. Photochemist. Photobiol. Sci. 1, 225–236. 10.1039/b201230h 12661961

[B108] Sponer J. Leszczynski J. Hobza P. (2001). Electronic properties, hydrogen bonding, stacking, and cation binding of DNA and RNA bases. Biopolymers 61, 3–31. 11891626 10.1002/1097-0282(2001)61:1<3::AID-BIP10048>3.0.CO;2-4

[B109] Suss-Fink G. (2010). Arene ruthenium complexes as anticancer agents. Dalton Trans. 39, 1673–1688. 10.1039/B916860P 20449402

[B110] Sviatenko L. Gorb L. Hovorun D. Leszczynski J. (2012). Interaction of 2-deoxyadenosine with cis-2-butene-1,4-dial: Computational approach to analysis of multistep chemical reactions. J. Phys. Chem. A 116, 2333–2342. 10.1021/jp211911u 22315946

[B111] Szabla R. Campos J. Sponer J. E. Sponer J. Gora R. W. Sutherland J. D. (2015). Excited-state hydrogen atom abstraction initiates the photochemistry of β-2′–deoxycytidine. Chem. Sci. 6, 2035–2043. 10.1039/C4SC03761H PMC4866440 27182431

[B112] Szolomajer J. Paragi G. Batta G. Guerra C. F. Bickelhaupt F. M. Kele Z. (2011). 3-substituted xanthines as promising candidates for quadruplex formation: computational, synthetic and analytical studies. New J. Chem. 35, 476–482. 10.1039/c0nj00612b

[B113] Terenzi A. Bonsignore R. Spinello A. Gentile C. Martorana A. Ducani C. (2014). Selective g-quadruplex stabilizers: schiff-base metal complexes with anticancer activity. RSC Adv. 4, 33245–33256. 10.1039/C4RA05355A

[B114] Valko M. Leibfritz D. Moncol J. Cronin M. T. Mazur M. Telser J. (2007). Free radicals and antioxidants in normal physiological functions and human disease. Int. J. Biochem. Cell Biol. 39, 44–84. 10.1016/j.biocel.2006.07.001 16978905

[B115] Vaya I. Brazard J. Gustavsson T. Markovitsi D. (2012). Electronically excited states of DNA oligonucleotides with disordered base sequences studied by fluorescence spectroscopy. Photochem. Photobiol. Sci. 11, 1767–1773. 10.1039/c2pp25180a 23034563

[B116] Vayá I. Gustavsson T. Douki T. Berlin Y. Markovitsi D. (2012). Electronic excitation energy transfer between nucleobases of natural DNA. J. Am. Chem. Soc. 134, 11366–11368. 10.1021/ja304328g 22765050

[B117] Very T. Ambrosek D. Otsuka M. Gourlaouen C. Assfeld X. Monari A. (2014). Photophysical properties of ruthenium(II) polypyridyl DNA intercalators: effects of the molecular surroundings investigated by theory. Chem. Euro. J. 20, 12901–12909. 10.1002/chem.201402963 25145959

[B118] Very T. Despax S. Hebraud P. Monari A. Assfeld X. (2012). Spectral properties of polypyridyl ruthenium complexes intercalated in DNA: theoretical insights into the surrounding effects of [Ru(dppz)(bpy)_2_]^2+^. Phys. Chem. Chem. Phys. 14, 12496–12504. 10.1039/c2cp40935f 22700035

[B119] Vianello R. Maksic Z. B. (2003). A combined ab initio and density functional study of the electronic structure of thymine and 2-thiothymine radicals. Collect. Czechoslovak Chem. Commun. 68, 2322–2334. 10.1135/cccc20032322

[B120] Vianello R. Repic M. Mavri J. (2012). How are biogenic amines metabolized by monoamine oxidases? Eur. J. Organ. Chem. 2012, 7057–7065. 10.1002/ejoc.201201122

[B121] Vorlickova M. Palecek E. (1974). A study of changes in DNA conformation caused by ionizing and ultra-violet radiation by means of pulse polarography and circular dichroism. Int. J. Radiat. Biol. 26, 363–372. 10.1080/09553007414551341 4548051

[B122] Wang K. Poon C. T. Choi C. Y. Wong W.-K. Kwong D. W. Yu F. Q. (2012). Synthesis, circular dichroism, DNA cleavage and singlet oxygen photogeneration of 4-amidinophenyl porphyrins. J. Porphyr. Phthalocyan. 16, 85–92. 10.1142/S108842461100435X

[B123] Wilhelm M. Mukherjee A. Bouvier B. Zakrzewska K. Hynes J. T. Lavery R. (2012). Multistep drug intercalation: molecular dynamics and free energy studies of the binding of daunomycin to DNA. J. Am. Chem. Soc. 134, 8588–8596. 10.1021/ja301649k 22548344

[B124] Wu Y. Mundy C. J. Colvin M. E. Car R. (2004). On the mechanisms of OH radical induced DNA-base damage: a comparative quantum chemical and CarParrinello molecular dynamics study. J. Phys. Chem. A 108, 2922–2929. 10.1021/jp0363592

[B125] Yadav A. Mishra P. C. (2013). Reactivities of hydroxyl and perhydroxyl radicals toward cytosine and thymine: a comparative study. Int. J. Quant. Chem. 113, 56–62. 10.1002/qua.24050

[B126] Yan Y. K. Melchart M. Habtemariam A. Sadler P. J. (2005). Organometallic chemistry, biology and medicine: ruthenium arene anticancer complexes. Chem. Commun. 4764–4776. 10.1039/b508531b 16193110

[B127] Zalesak J. Lourdin M. Krejci L. Constant J.-F. Jourdan M. (2014). Structure and dynamics of DNA duplexes containing a cluster of mutagenic 8-oxoguanine and abasic site lesions. J. Mol. Biol. 426, 1524–1538. 10.1016/j.jmb.2013.12.022 24384094

[B128] Zeglis B. M. Pierre V. C. Barton J. K. (2007). Metallo-intercalators and metallo-insertors. Chem. Commun. 4565–4579. 10.1039/b710949k PMC2790054 17989802

[B129] Zinovjev K. Ruiz-Pernia J. J. Tunon I. (2013). Toward an automatic determination of enzymatic reaction mechanisms and their activation free energies. J. Chem. Theory Comput. 9, 3740–3749. 10.1021/ct400153r 26584125

[B130] Zinovjev K. Tunon I. (2014). Exploring chemical reactivity of complex systems with path-based coordinates: role of the distance metric. J. Comput. Chem. 35, 1672–1681. 10.1002/jcc.23673 24986052

